# Supplementary figures and images for: Robust membrane protein tweezers reveal the folding speed limit of helical membrane proteins
Source: eLife. 2023 May 30;12:e85882. doi: 10.7554/eLife.85882 (PMC10259496; doi:10.7554/eLife.85882)

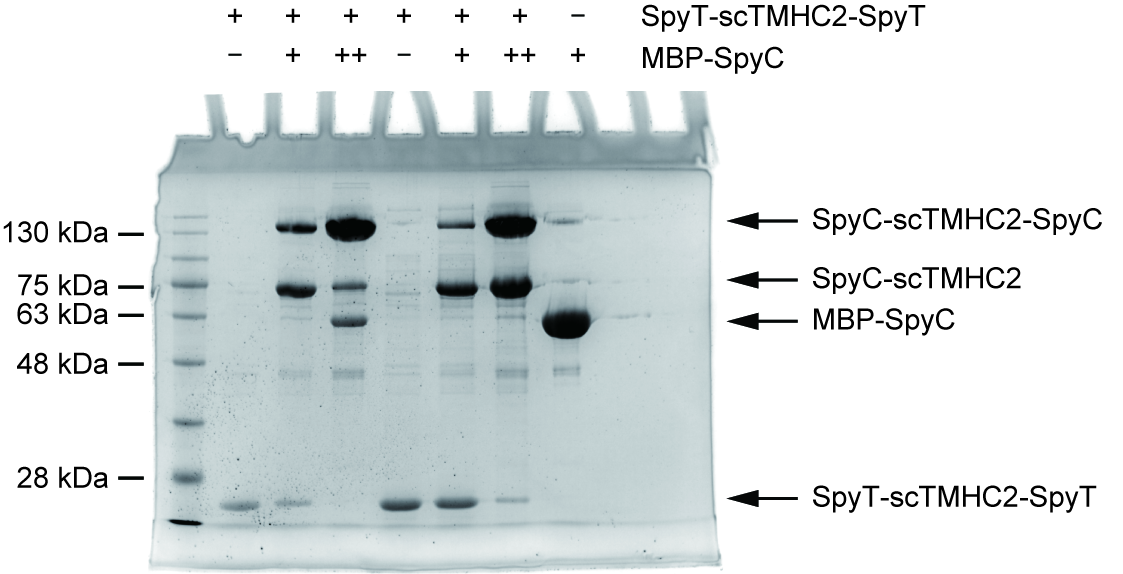

Supplement: Figure 1—source data 1. [file elife-85882-fig1-data1.zip › Figures with uncropped gel B.tif]

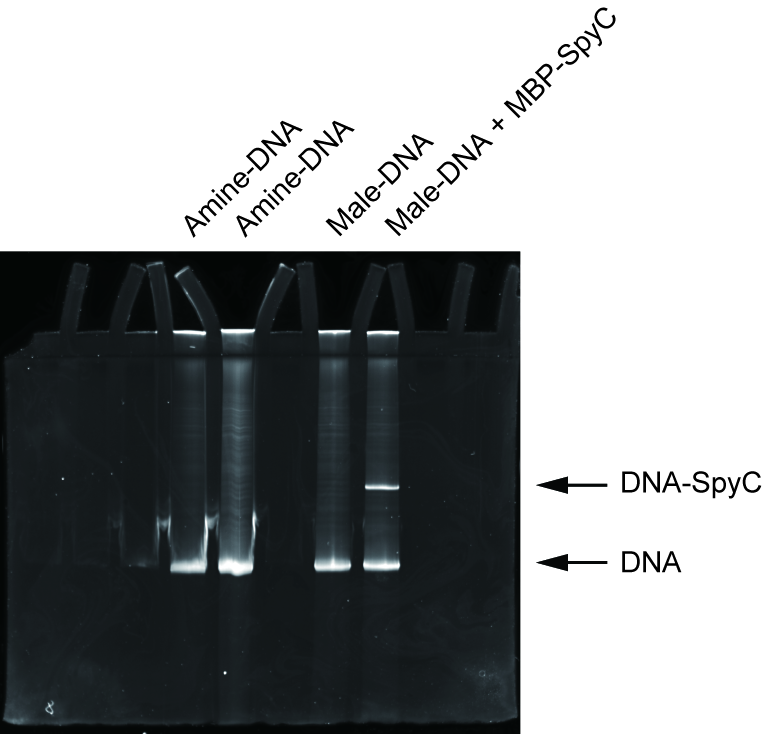

Supplement: Figure 1—source data 1. [file elife-85882-fig1-data1.zip › Figures with uncropped gel C.tif]

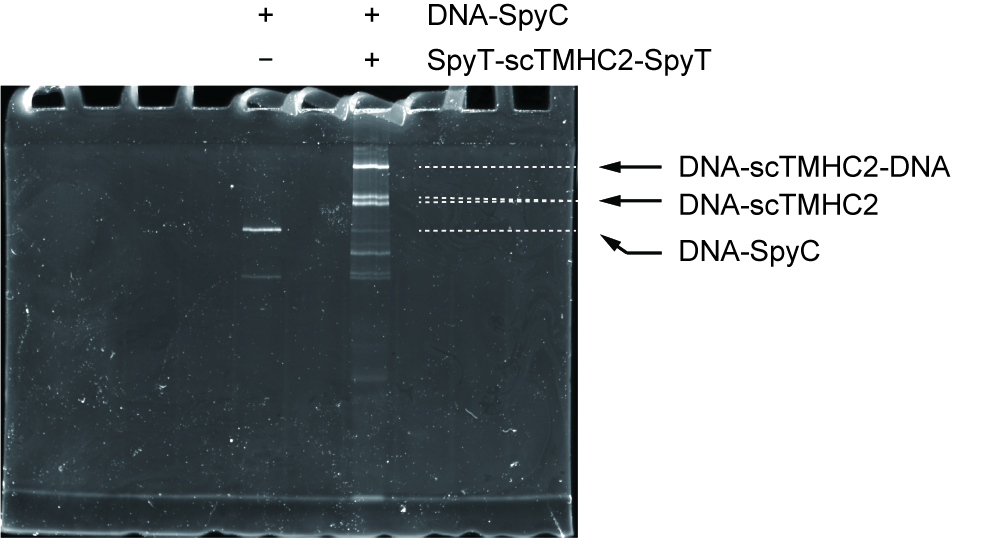

Supplement: Figure 1—source data 1. [file elife-85882-fig1-data1.zip › Figures with uncropped gel D.tif]

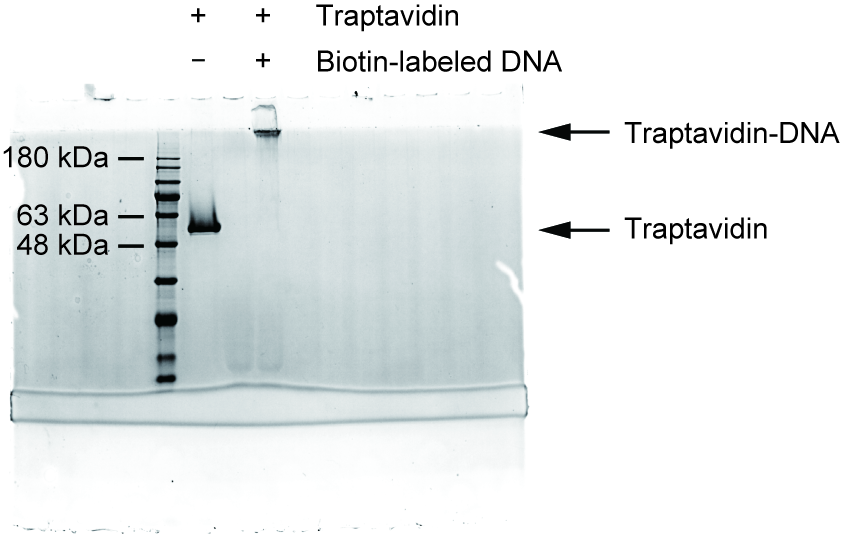

Supplement: Figure 1—source data 1. [file elife-85882-fig1-data1.zip › Figures with uncropped gel E.tif]

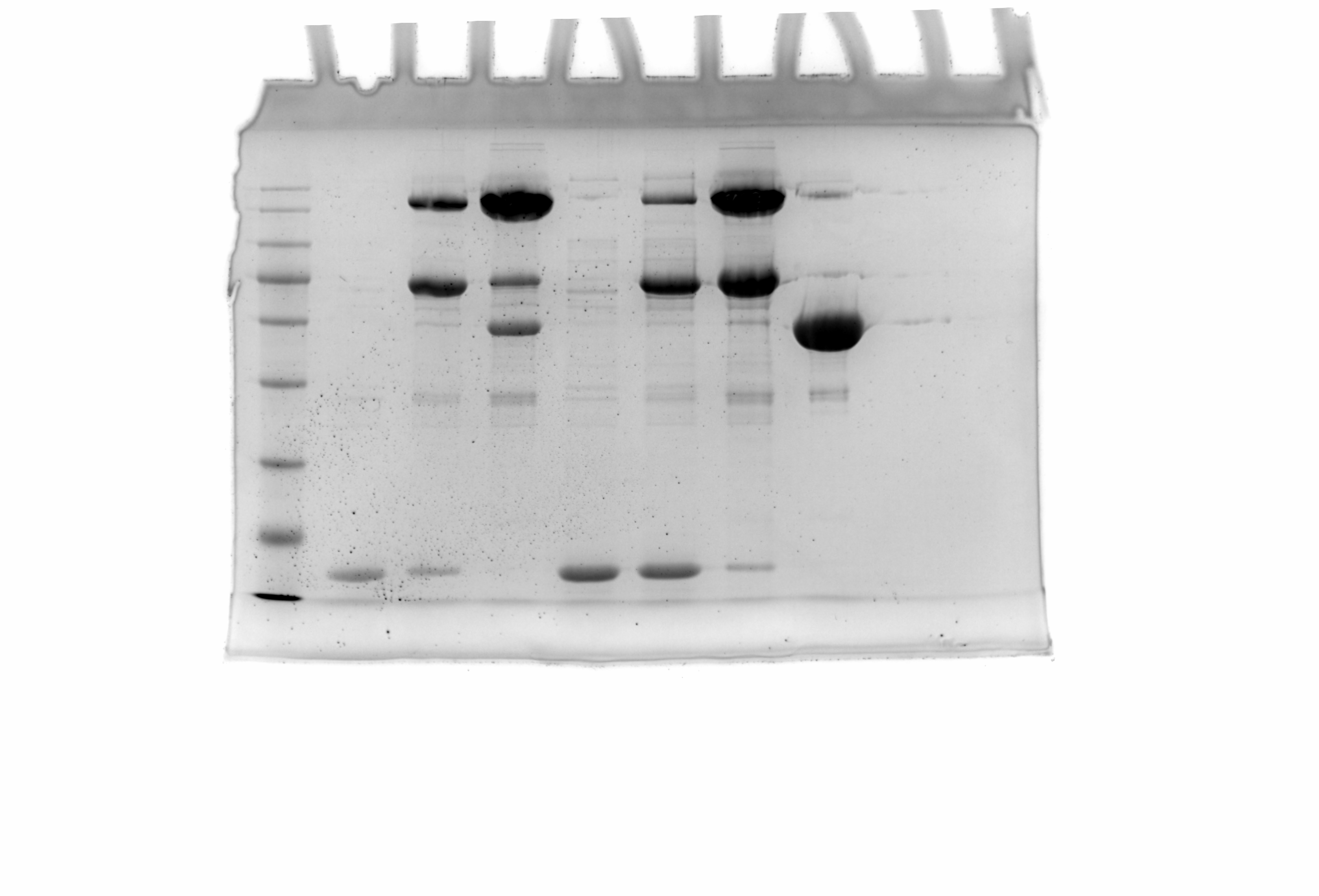

Supplement: Figure 1—source data 1. [file elife-85882-fig1-data1.zip › Original file - gel B.tif]

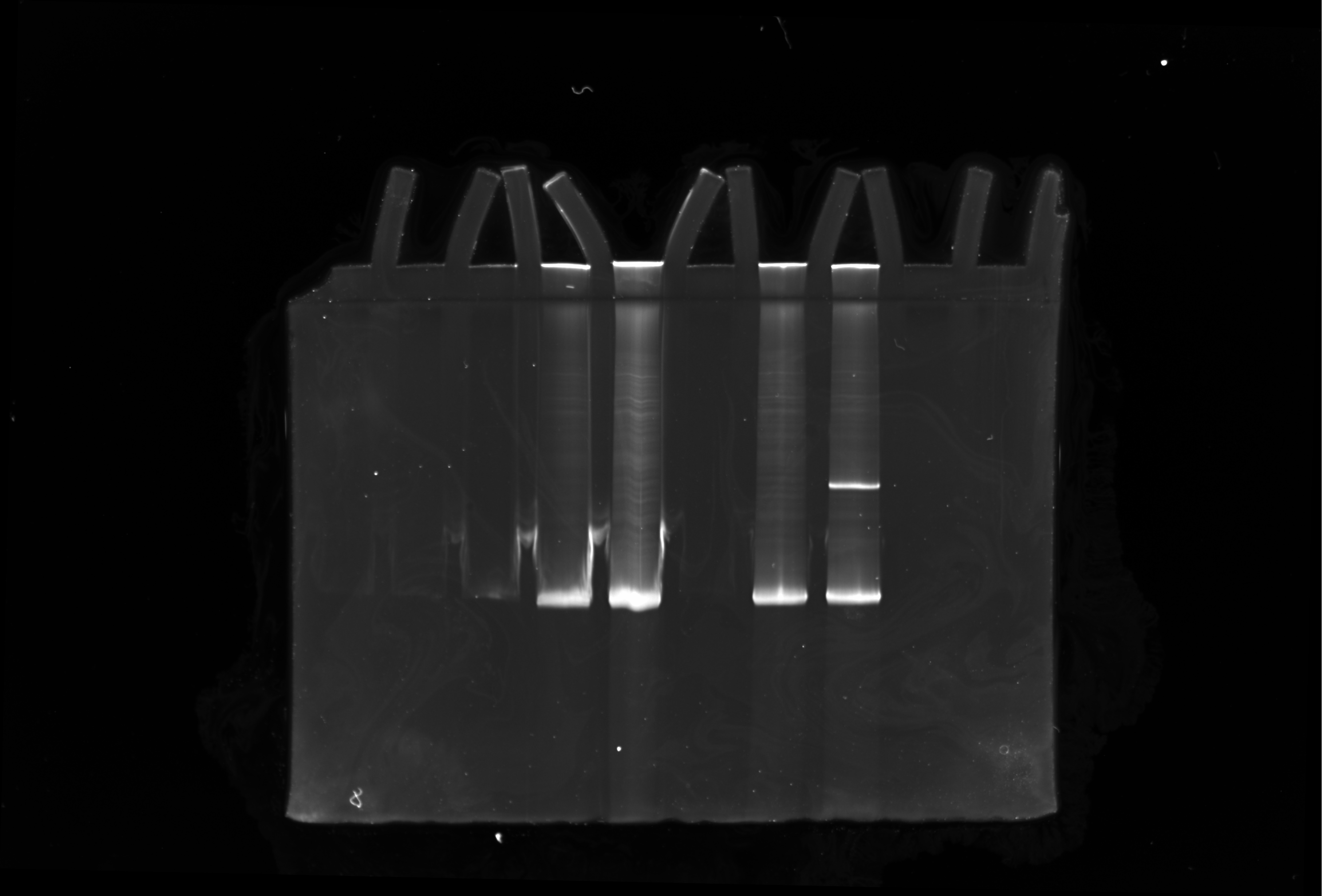

Supplement: Figure 1—source data 1. [file elife-85882-fig1-data1.zip › Original file - gel C.tif]

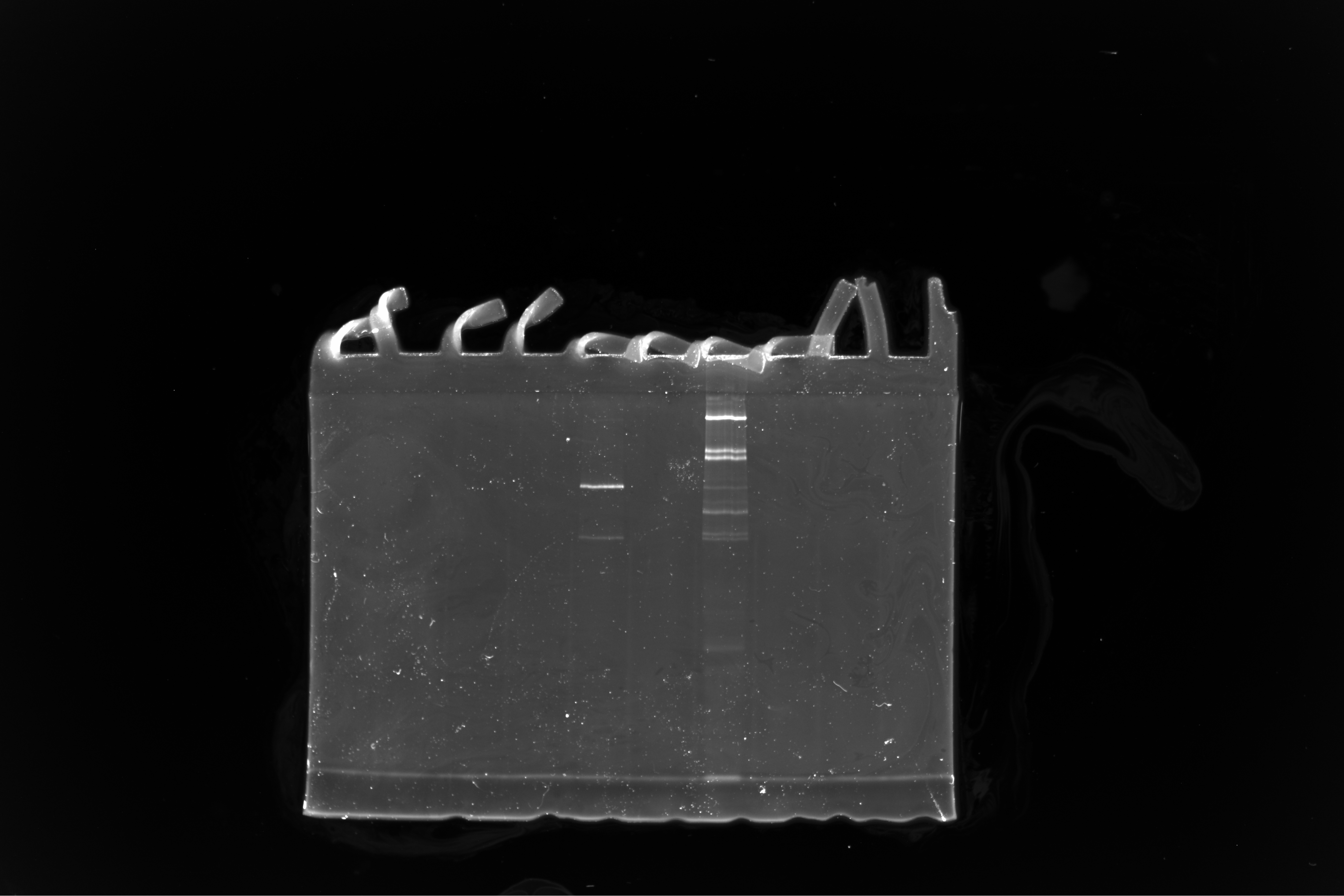

Supplement: Figure 1—source data 1. [file elife-85882-fig1-data1.zip › Original file - gel D.tif]

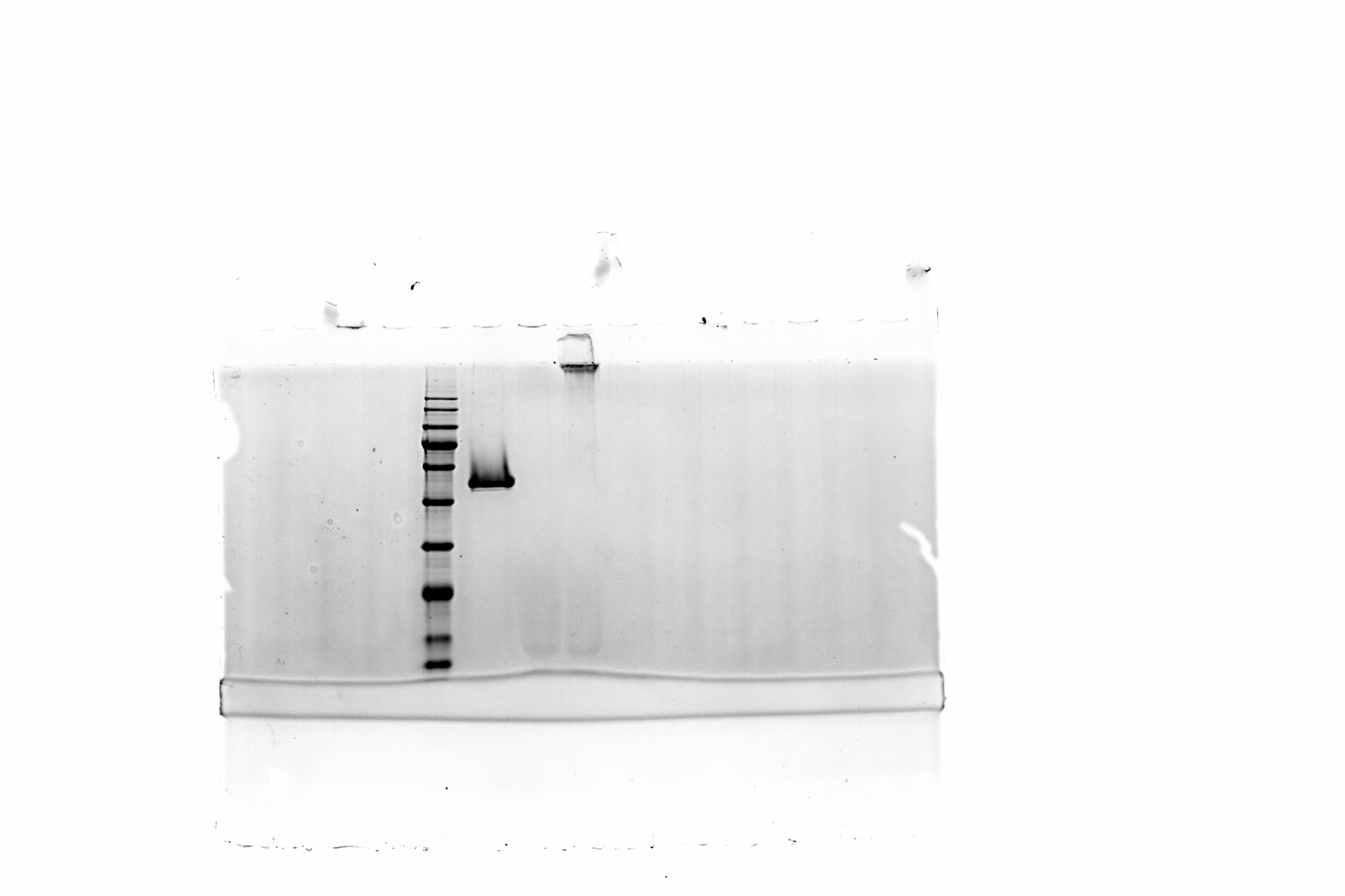

Supplement: Figure 1—source data 1. [file elife-85882-fig1-data1.zip › Original file - gel E.tif]
