# Supplementary material for: Robust membrane protein tweezers reveal the folding speed limit of helical membrane proteins
Source: eLife. 2023 May 30;12:e85882. doi: 10.7554/eLife.85882 (PMC10259496; doi:10.7554/eLife.85882)
Supplement: Figure 3—source data 1. [file elife-85882-fig3-data1.zip › Full list of tested conditions for DBCO-azide conjugation.docx]

|  | DBCO^a^  (mM) | DNA handle  (bp) | Buffer composition^b^ | Target molecule^c^ (pM) | Conjugation^d^ |
| --- | --- | --- | --- | --- | --- |
| Membrane protein scTMHC2 | 0.025 | 512 | PBS | 50 | O |
|  |  |  | 0.005% DDM, TBS |  | X |
|  |  |  | 0.1% DDM, TBS |  | X |
|  |  |  | 0.1% DDM, PBS |  | X |
|  |  |  | 0.1% DDM, 1% glycerol, PBS |  | X |
|  | 0.1 |  | PBS | 20 | O |
|  |  |  |  | 50 | O |
|  |  |  |  | 100 | O |
|  |  |  | 0.001% DDM, PBS | 100 | X |
|  |  |  | 0.1% DDM, PBS | 35 | X |
|  |  |  |  | 50 | X |
|  |  |  |  | 100 | X |
|  |  |  | 0.1% DDM, TBS | 5 | X |
|  |  |  |  | 10 | X |
|  |  |  |  | 20 | X |
|  |  |  |  | 40 | X |
|  |  |  |  | 50 | X |
|  |  |  | 0.1% CHAPSO, PBS | 100 | X |
|  |  |  | 1% glycerol, PBS | 100 | X |
|  |  |  | 1% bicelle (C), TBS | 20 | X |
|  |  |  |  | 40 | X |
|  |  |  | 1.5% bicelle (C), TBS | 10 | X |
|  |  |  |  | 20 | X |
|  |  |  |  | 40 | X |
|  |  | 1022 | PBS | 20 | O |
|  |  |  |  | 100 | O |
|  |  |  |  | 300 | O |
|  |  |  | TBS | 200 | O |
|  |  |  | 0.0001% DDM, PBS | 300 | O |
|  |  |  | 0.01% DDM, TBS | 200 | O |
|  |  |  | 0.05% DDM, TBS | 200 | O |
|  |  |  | 0.1% DDM, TBS | 300 | X |
|  |  |  | 0.1% DDM, PBS | 300 | X |
|  |  |  | 0.1% lyso PC (14:0), TBS | 150 | X |
|  |  |  | 0.5% bicelle (C), TBS | 150 | X |
|  |  |  |  | 200 | X |
|  |  |  | 1% bicelle (C), TBS | 150 | X |
|  |  |  |  | 300 | X |
|  |  |  | 1.5% bicelle (C), TBS | 150 | X |
|  |  |  | 2% bicelle (C), TBS | 150 | X |
|  |  |  |  | 300 | X |
|  |  |  | 2% bicelle (G), TBS | 150 | X |
|  | 1 | 512 | PBS | 100 | X |
|  |  |  | 2% bicelle (C), PBS | 100 | X |
| DNA hairpin 17S6L | 0.01 | 2011 | PBS | 20 | X |
|  |  |  |  | 50 | X |
|  |  |  |  | 100 | X |
|  | 0.025 |  | PBS | 50 | O |
|  |  |  |  | 100 | O |
|  |  |  | 0.1% DDM, PBS | 50 | O |
|  |  |  | 1.5% bicelle (C), PBS | 50 | X |
|  | 0.05 |  | PBS | 50 | O |
|  | 0.075 |  |  | 100 | O |
|  | 0.1 | 512 |  | 30 | X |
|  |  | 1024 |  | 30 | O |
|  |  | 2011 |  | 10 | O |
|  |  |  |  | 20 | O |
|  |  |  |  | 40 | O |
|  |  |  | 0.1% DDM, PBS | 20 | O |
|  |  |  |  | 40 | O |
|  |  |  | 1% bicelle (C), PBS | 100 | O |
|  |  |  | 1% bicelle (C), TBS | 40 | O |
|  |  |  | 1.5% bicelle (C), PBS | 100 | O |

Figure 3–source data 1. Full list of tested conditions for DBCO-azide conjugation. ^a^ DBCO concentration indicates the final concentration of DBCO-sulfo-NHS crosslinker of DBCO modification on bead surface. The other conditions (DNA handle length, buffer composition, and targe molecule concentration) indicate those in DBCO-azide conjugation step of the single-molecule system assembly. ^b^ PBS indicates a phosphate-buffered saline (0.1 M sodium phosphate, 150 mM NaCl, pH 7.3). TBS indicates a Tris-buffered saline (50 mM Tris-HCl, 150 mM NaCl, pH 7.5). The bicelle (C or G) consists of DMPC (or DMPG) lipid and CHAPSO detergent at a 2.5:1 molar ratio. The % indicates w/v %. ^c^ Final concentration of target molecule (membrane protein or DNA hairpin) for the surface tethering. ^d^ The symbol O indicates successful conjugation between DBCO-modified beads and target membrane proteins. In this case, specifically-tethered beads are found and the force-extension curves of the molecular constructs are obtained (confirmed by three replicates). The symbol X indicates unsuccessful conjugation or entire nonspecific binding of beads to the surface. In this case, no data are obtained. See Figure 3–source data 1 for the full list.
