# Supplementary material for: Robust membrane protein tweezers reveal the folding speed limit of helical membrane proteins
Source: eLife. 2023 May 30;12:e85882. doi: 10.7554/eLife.85882 (PMC10259496; doi:10.7554/eLife.85882)
Supplement: Figure 4—source data 1. [file elife-85882-fig4-data1.zip › Survey of protein unfolding forces.docx]

| Protein | Unfolding force (pN)^a^ | Force-loading rate (pN/s) | Molecular weight (kDa) | Method^b^ |
| --- | --- | --- | --- | --- |
| LZ26 zipper^1^ | 15^c^ | 125 | 11.3 | OT |
| Barnase^2^ | 20^c^ | 4.1 | 12.4 | OT |
| UCH-L1^3^ | 37^c^ | 70 | 24.5 | OT |
| vWF multimer^4^  vWF (A1A2A3)_3_ | 22.8, 40.0  23.1 | 90  22 | 250  210 | OT |
| cSc^5^ | 8.5, 8.9, 9.8  11.3, 11.4 | 1.5, 3.2, 5.5  8.6, 15.1 | 24.2 | OT |
| HIV-1-PR^6^ | 23.0, 24.2, 40.5 | 5 | 10.7 | OT |
| T4L (16, 61)^7^  T4L (16, 159) | 51.8  47.9 | 4.5  13.5 | 5.0  16.2 | OT |
| MJ0336^8^ | 19.5^c^ | 10 | 10.0 | OT |
| Calmodulin^9^ | 21^c^ | 5.5 | 16.8 | OT |
| ACBP (1, 86)^10^  ACBP (46, 86) | 10.7, 10.9  18 | 15, 45  11 | 9.9  4.8 | OT |
| PrP^11^ | 10 | 37.5 | 16.2 | OT |
| Apo-Mb (1, 153)^12^  Apo-Mb (53, 153) | 12.5  12.0 | 5 | 17.2  11.2 | OT |
| RNase H^13^ | 19 | 13,53 | 17.5 | OT |
| NCS1^14^ | 10.6 (N-domain)  13.8 (C-domain) | 5 | 21.4 | OT |
| Apo-SOD1^15^ | 10^c^ | 91 | 15.8 | OT |
| MBP^16^ | 25^c^ | 5 | 40.7 | OT |
| Luciferase^17^ | 40^c^ | 8.5 | 60.7 | OT |
| CBD-A^18^ | 9^c^ | 5 | 15.2 | OT |
| Hsp90^19^ | 14.1 (N-domain)  17.6 (M-domain)  9.2 (C-domain) | 4 | 81.3 | OT |
| EF-G (full)^20^  EF-G (G-II-III) | 36^c^  38^c^ | 10 | 77.6  53.8 | OT |
| DHFR^21^ | 26^c^ | 6 | 21.5 | OT |
| PKA^22^ | 10.5, 13.6 (CNB-A)  5.2, 9.1 (CNB-B) | 5.6 | 28.9 | OT |
| pARC^23^ | 9.0 | 7.5 | 11.7 | OT |
| Arc-L1-Arc (unknotted)^23^  Arc-L1-Arc (knotted) | 8.2  14.7 | 7.5 | 13.2 | OT |
| ROSS^24^ | 17 | 100 | 12.4 | OT |
| NuG2^25^ | 34^c^ | 2.55 | 6.2 | OT |
| RTX (1529, 1664)^26^  RTX (1529, 1681) | 12.7  14.2 | 8.5 | 16.0 | OT |
| Top7^27^ | 42 | 7.5 | 10.6 | OT |
| Vim2B-N^28^  Vim2B-C | 8^c^  10^c^ | 137.5 | 20.8  20.7 | OT |
| FLNa20^29^ | 15 | 55 | 29.6 | OT |
| FL domain^30^ | 5^c^ | 40, 200 | 9.7 | OT |
| PADC^31^ | 15 | 8.5 | 17.6 | OT |
| AFV3-109^32^ | 10 | 5 | 12.3 | OT |
| GFP^33^ | 40^c^ | 210 | 26.6 | OT |
| Syt1 C2AB^34^  E-Syt2 C2AB  E-Syt2 C2C | 12^c^, 28^c^  16^c^, 32^c^  27^c^ | 1.5 | 37.2  35.0  17.0 | OT |
| SNARE complex (Vn)^35^  SNARE complex (Vc) | 16.7^c^  18.5^c^ | 0.6 | 41.7 | OT |
| SNARE complex^36^ | 14.4 | 1 | 42.8 | MT |
| Spectrin^37^ | 10.8, 15.7,  26.1, 30.0, 33.2 | 0.05, 0.1,  0.5, 1.0, 2.0 | 61.0 | MT |
| scTMHC2^38^ | 18^c^ | 0.5^d^ | 17.8 | MT |
| GlpG^39,40^ | 22.5^c^, 25^c^ | 1, 0.5^d^ | 21.3 | MT |
| β_2_AR^40^ | 14.2^c^ | 1 | 39.6 | MT |
| ClC^41^ | 46^c^ | 0.5^d^ | 47.5 | MT |

Figure 4–source data 1. Protein unfolding forces measured by single-molecule tweezers. ^a^ The unfolding forces indicate the most probable unfolding forces except the items with the superscript c. ^b^ OT and MT indicate optical tweezers and magnetic tweezers, respectively. ^c^ The unfolding force is an approximate or averaged value. ^d^ The force-loading rate is an averaged value over the force scanning of 1–50 pN.

References

1 Gebhardt, J. C., Bornschlogl, T. & Rief, M. Full distance-resolved folding energy landscape of one single protein molecule. *Proc Natl Acad Sci U S A* **107**, 2013-2018, doi:10.1073/pnas.0909854107 (2010).

2 Alemany, A., Rey-Serra, B., Frutos, S., Cecconi, C. & Ritort, F. Mechanical Folding and Unfolding of Protein Barnase at the Single-Molecule Level. *Biophys J* **110**, 63-74, doi:10.1016/j.bpj.2015.11.015 (2016).

3 Ziegler, F. *et al.* Knotting and unknotting of a protein in single molecule experiments. *Proc Natl Acad Sci U S A* **113**, 7533-7538, doi:10.1073/pnas.1600614113 (2016).

4 Ying, J., Ling, Y., Westfield, L. A., Sadler, J. E. & Shao, J. Y. Unfolding the A2 domain of von Willebrand factor with the optical trap. *Biophys J* **98**, 1685-1693, doi:10.1016/j.bpj.2009.12.4324 (2010).

5 Zhang, X. F., Zhang, W., Quach, M. E., Deng, W. & Li, R. Force-Regulated Refolding of the Mechanosensory Domain in the Platelet Glycoprotein Ib-IX Complex. *Biophys J* **116**, 1960-1969, doi:10.1016/j.bpj.2019.03.037 (2019).

6 Caldarini, M. *et al.* The complex folding behavior of HIV-1-protease monomer revealed by optical-tweezer single-molecule experiments and molecular dynamics simulations. *Biophys Chem* **195**, 32-42, doi:10.1016/j.bpc.2014.08.001 (2014).

7 Shank, E. A., Cecconi, C., Dill, J. W., Marqusee, S. & Bustamante, C. The folding cooperativity of a protein is controlled by its chain topology. *Nature* **465**, 637-640, doi:10.1038/nature09021 (2010).

8 Ramirez, M. P. *et al.* Single molecule force spectroscopy reveals the effect of BiP chaperone on protein folding. *Protein Sci* **26**, 1404-1412, doi:10.1002/pro.3137 (2017).

9 Yu, Z., Cui, Y., Selvam, S., Ghimire, C. & Mao, H. Dissecting cooperative communications in a protein with a high-throughput single-molecule scalpel. *Chemphyschem* **16**, 223-232, doi:10.1002/cphc.201402443 (2015).

10 Heidarsson, P. O. *et al.* A highly compliant protein native state with a spontaneous-like mechanical unfolding pathway. *J Am Chem Soc* **134**, 17068-17075, doi:10.1021/ja305862m (2012).

11 Gupta, A. N. *et al.* Pharmacological chaperone reshapes the energy landscape for folding and aggregation of the prion protein. *Nature Communications* **7**, 12058, doi:10.1038/ncomms12058 (2016).

12 Elms, P. J., Chodera, J. D., Bustamante, C. & Marqusee, S. The molten globule state is unusually deformable under mechanical force. *Proceedings of the National Academy of Sciences* **109**, 3796-3801, doi:10.1073/pnas.1115519109 (2012).

13 Cecconi, C., Shank, E. A., Bustamante, C. & Marqusee, S. Direct observation of the three-state folding of a single protein molecule. *Science* **309**, 2057-2060, doi:10.1126/science.1116702 (2005).

14 Heidarsson, P. O. *et al.* Single-molecule folding mechanism of an EF-hand neuronal calcium sensor. *Structure* **21**, 1812-1821, doi:10.1016/j.str.2013.07.022 (2013).

15 Sen Mojumdar, S. *et al.* Partially native intermediates mediate misfolding of SOD1 in single-molecule folding trajectories. *Nat Commun* **8**, 1881, doi:10.1038/s41467-017-01996-1 (2017).

16 Bechtluft, P. *et al.* Direct Observation of Chaperone-Induced Changes in a Protein Folding Pathway. *Science* **318**, 1458-1461, doi:10.1126/science.1144972 (2007).

17 Mashaghi, A., Mashaghi, S. & Tans, S. J. Misfolding of luciferase at the single-molecule level. *Angew Chem Int Ed Engl* **53**, 10390-10393, doi:10.1002/anie.201405566 (2014).

18 Hao, Y., Canavan, C., Taylor, S. S. & Maillard, R. A. Integrated Method to Attach DNA Handles and Functionally Select Proteins to Study Folding and Protein-Ligand Interactions with Optical Tweezers. *Sci Rep* **7**, 10843, doi:10.1038/s41598-017-11214-z (2017).

19 Jahn, M. *et al.* The charged linker of the molecular chaperone Hsp90 modulates domain contacts and biological function. *Proc Natl Acad Sci U S A* **111**, 17881-17886, doi:10.1073/pnas.1414073111 (2014).

20 Liu, K., Chen, X. & Kaiser, C. M. Energetic dependencies dictate folding mechanism in a complex protein. *Proc Natl Acad Sci U S A* **116**, 25641-25648, doi:10.1073/pnas.1914366116 (2019).

21 Wruck, F., Katranidis, A., Nierhaus, K. H., Buldt, G. & Hegner, M. Translation and folding of single proteins in real time. *Proc Natl Acad Sci U S A* **114**, E4399-E4407, doi:10.1073/pnas.1617873114 (2017).

22 England, J. P. *et al.* Switching of the folding-energy landscape governs the allosteric activation of protein kinase A. *Proc Natl Acad Sci U S A* **115**, E7478-E7485, doi:10.1073/pnas.1802510115 (2018).

23 Bustamante, A. *et al.* The energy cost of polypeptide knot formation and its folding consequences. *Nat Commun* **8**, 1581, doi:10.1038/s41467-017-01691-1 (2017).

24 Mehlich, A., Fang, J., Pelz, B., Li, H. & Stigler, J. Slow Transition Path Times Reveal a Complex Folding Barrier in a Designed Protein. *Front Chem* **8**, 587824, doi:10.3389/fchem.2020.587824 (2020).

25 Lei, H. *et al.* Single-Molecule Force Spectroscopy Trajectories of a Single Protein and Its Polyproteins Are Equivalent: A Direct Experimental Validation Based on A Small Protein NuG2. *Angew Chem Int Ed Engl* **56**, 6117-6121, doi:10.1002/anie.201610648 (2017).

26 Wang, H., Gao, X. & Li, H. Single Molecule Force Spectroscopy Reveals the Mechanical Design Governing the Efficient Translocation of the Bacterial Toxin Protein RTX. *J Am Chem Soc* **141**, 20498-20506, doi:10.1021/jacs.9b11281 (2019).

27 Goldman, D. H. *et al.* Ribosome. Mechanical force releases nascent chain-mediated ribosome arrest in vitro and in vivo. *Science* **348**, 457-460, doi:10.1126/science.1261909 (2015).

28 Ramm, B. *et al.* Sequence-resolved free energy profiles of stress-bearing vimentin intermediate filaments. *Proc Natl Acad Sci U S A* **111**, 11359-11364, doi:10.1073/pnas.1403122111 (2014).

29 Rognoni, L., Most, T., Zoldak, G. & Rief, M. Force-dependent isomerization kinetics of a highly conserved proline switch modulates the mechanosensing region of filamin. *Proc Natl Acad Sci U S A* **111**, 5568-5573, doi:10.1073/pnas.1319448111 (2014).

30 Fang, J. *et al.* Forced protein unfolding leads to highly elastic and tough protein hydrogels. *Nat Commun* **4**, 2974, doi:10.1038/ncomms3974 (2013).

31 Wang, H. *et al.* Mechanical Unfolding and Folding of a Complex Slipknot Protein Probed by Using Optical Tweezers. *Biochemistry* **58**, 4751-4760, doi:10.1021/acs.biochem.9b00320 (2019).

32 He, C. *et al.* Direct observation of the fast and robust folding of a slipknotted protein by optical tweezers. *Nanoscale* **11**, 3945-3951, doi:10.1039/c8nr10070e (2019).

33 Ganim, Z. & Rief, M. Mechanically switching single-molecule fluorescence of GFP by unfolding and refolding. *Proc Natl Acad Sci U S A* **114**, 11052-11056, doi:10.1073/pnas.1704937114 (2017).

34 Ma, L. *et al.* Single-molecule force spectroscopy of protein-membrane interactions. *Elife* **6**, doi:10.7554/eLife.30493 (2017).

35 Gao, Y. *et al.* Single reconstituted neuronal SNARE complexes zipper in three distinct stages. *Science* **337**, 1340-1343, doi:10.1126/science.1224492 (2012).

36 Shon, M. J., Kim, H. & Yoon, T. Y. Focused clamping of a single neuronal SNARE complex by complexin under high mechanical tension. *Nat Commun* **9**, 3639, doi:10.1038/s41467-018-06122-3 (2018).

37 Renn, J. P. *et al.* Mechanical unfolding of spectrin reveals a super-exponential dependence of unfolding rate on force. *Sci Rep* **9**, 11101, doi:10.1038/s41598-019-46525-w (2019).

38 Lu, P. *et al.* Accurate computational design of multipass transmembrane proteins. *Science* **359**, 1042-1046, doi:10.1126/science.aaq1739 (2018).

39 Min, D., Jefferson, R. E., Bowie, J. U. & Yoon, T. Y. Mapping the energy landscape for second-stage folding of a single membrane protein. *Nat Chem Biol* **11**, 981-987, doi:10.1038/nchembio.1939 (2015).

40 Choi, H. K. *et al.* Watching helical membrane proteins fold reveals a common N-to-C-terminal folding pathway. *Science* **366**, 1150-1156, doi:10.1126/science.aaw8208 (2019).

41 Min, D. *et al.* Unfolding of a ClC chloride transporter retains memory of its evolutionary history. *Nat Chem Biol* **14**, 489-496, doi:10.1038/s41589-018-0025-4 (2018).
