# Supplementary material for: Robust membrane protein tweezers reveal the folding speed limit of helical membrane proteins
Source: eLife. 2023 May 30;12:e85882. doi: 10.7554/eLife.85882 (PMC10259496; doi:10.7554/eLife.85882)
Supplement: Figure 7—source data 1. [file elife-85882-fig7-data1.zip › Survey of measured viscosity for DMPC lipid membranes.docx]

Figure 7–source data 1. Survey of measured viscosity for DMPC lipid membranes

| Membrane system^a^ | Temperature (K) | Measurement method | Membrane viscosity (*η*_m_, mPa·s)^b^ | Diffusion coefficient (*D*_m_, μm^2^/s)^c^ | Viscosity ratio  (*η*_m_/*η*_w_) |
| --- | --- | --- | --- | --- | --- |
| SUV | 298 | Neutron spin echo spectroscopy^1^ | ~20113^d^ |  | 22599 |
| SUV | 298 | Picosecond time-resolved fluorescence spectroscopy^2^ | 97^e^ |  | 109 |
| SUV | 291 | Picosecond time-resolved fluorescence spectroscopy^2^ | ~1200^e^ |  | 1200 |
| LUV | 295 | Fluorescence lifetime spectroscopy and imaging^3^ | ~280 |  | 280 |
| GUV | 298 | Optical dynamometry^4^ | 3486^d^ |  | 3917 |
| MLV | 298 | Electron spin resonance^5^ | 223, 64, 63^f^ |  | 250, 72, 70 |
| SLB | 298 | Pulsed field gradient ^1^H NMR^6^ |  | 5.70 | 404^g^ |

^a^ SUV, LUV, GUV, MLV, and SLB indicate the small unilamellar vesicle, large unilamellar vesicle, giant unilamellar vesicle, multilamellar vesicle, and supported lipid bilayer, respectively. ^b^ For comparison, the dynamic viscosity of water (*η*_w_) is approximately 0.89 mPa·s at 298K. ^c^ For comparison, the diffusion coefficient of water (*D*_w_) is approximately 2.3$\times$10^3^ μm^2^/s at 298K. ^d^ The surface shear viscosity (*η*_s_) in the original reference was converted to the bulk membrane viscosity (*η*_m_) by the relation *η*_s_ = *η*_m_*h* (ref. ^4^), where *h* is the DMPC membrane thickness of ~3.7 nm for SUV and MLV (refs. ^7,8^). ^e^ The values are those from the later steady-state lifetimes in fluorescence decay curves. ^f^ The values are those measured at three different bilayer depths. ^g^ The viscosity ratio was calculated using the Einstein relation.

References

1 Nagao, M., Kelley, E. G., Ashkar, R., Bradbury, R. & Butler, P. D. Probing Elastic and Viscous Properties of Phospholipid Bilayers Using Neutron Spin Echo Spectroscopy. *J Phys Chem Lett* **8**, 4679-4684, doi:10.1021/acs.jpclett.7b01830 (2017).

2 Nojima, Y. & Iwata, K. Viscosity Heterogeneity inside Lipid Bilayers of Single-Component Phosphatidylcholine Liposomes Observed with Picosecond Time-Resolved Fluorescence Spectroscopy. *J Phys Chem B* **118**, 8631-8641, doi:10.1021/jp503921e (2014).

3 Wu, Y. L. *et al.* Molecular rheometry: direct determination of viscosity in L-o and L-d lipid phases via fluorescence lifetime imaging. *Phys Chem Chem Phys* **15**, 14986-14993, doi:10.1039/c3cp51953h (2013).

4 Dimova, R., Pouligny, B. & Dietrich, C. Pretransitional effects in dimyristoylphosphatidylcholine vesicle membranes: Optical dynamometry study. *Biophys J* **79**, 340-356, doi:Doi 10.1016/S0006-3495(00)76296-5 (2000).

5 Bahri, M. A. *et al.* Quantification of lipid bilayer effective microviscosity and fluidity effect induced by propofol. *Biophys Chem* **114**, 53-61, doi:10.1016/j.bpc.2004.11.006 (2005).

6 Filippov, A., Oradd, G. & Lindblom, G. Influence of cholesterol and water content on phospholipid lateral diffusion in bilayers. *Langmuir* **19**, 6397-6400, doi:10.1021/la034222x (2003).

7 Kucerka, N., Nieh, M. P. & Katsaras, J. Fluid phase lipid areas and bilayer thicknesses of commonly used phosphatidylcholines as a function of temperature. *Bba-Biomembranes* **1808**, 2761-2771, doi:10.1016/j.bbamem.2011.07.022 (2011).

8 Nagle, J. F. & Tristram-Nagle, S. Structure of lipid bilayers. *Bba-Rev Biomembranes* **1469**, 159-195, doi:Doi 10.1016/S0304-4157(00)00016-2 (2000).
